# Antibacterial and Antifungal Properties of a Novel Antimicrobial Peptide GK-19 and Its Application in Skin and Soft Tissue Infections Induced by *MRSA* or *Candida albicans*

**DOI:** 10.3390/pharmaceutics14091937

**Published:** 2022-09-13

**Authors:** Chenghua Song, Ruichao Wen, Jiaxuan Zhou, Xiaoyan Zeng, Zi Kou, Jia Zhang, Tao Wang, Pengkang Chang, Yi Lv, Rongqian Wu

**Affiliations:** 1 National Local Joint Engineering Research Center for Precision Surgery & Regenerative Medicine, Shaanxi Provincial Center for Regenerative Medicine and Surgical Engineering, The First Affiliated Hospital of Xi’an Jiaotong University, Xi’an 710061, China; 2 Department of Laboratory Medicine, The First Affiliated Hospital of Xi’an Jiaotong University, Xi’an 710061, China; 3 Department of Gastroenterology, The Second Affiliated Hospital of Xi’an Jiaotong University, Xi’an 710004, China; 4 Department of Hepatobiliary Surgery, The First Affiliated Hospital of Xi’an Jiaotong University, Xi’an 710061, China

**Keywords:** antimicrobial peptides, GK-19, skin and soft tissue infections, *MRSA*, *Candida albicans*

## Abstract

The increasing resistance of human pathogens promotes the development of novel antimicrobial agents. Due to the physical bactericidal mechanism of membrane disruption, antimicrobial peptides are considered as potential therapeutic candidates without inducing microbial resistance. Scorpion venom-derived peptide, *Androctonus amoreuxi* Antimicrobial Peptide 1 (AamAP1), has been proved to have broad-spectrum antimicrobial properties. However, AamAP1 can induce hemolysis and shows strong toxicity against mammalian cells. Herein, the antimicrobial activity and mechanism of a novel synthetic antimicrobial peptide, GK-19, derived from AamAP1 and its derivatives, was evaluated. Five bacteria and three fungi were used to evaluate the antimicrobial effects of GK-19 in vitro. Scalded mice models combined with skin and soft tissue infections (SSTIs) were used to evaluate its applicability. The results indicated that GK-19 could not only inhibit Gram-positive and Gram-negative bacterial growth, but also kill fungi by disrupting the microbial cell membrane. Meanwhile, GK-19 showed negligible toxicity to mammalian cells, low hemolytic activity and high stability in plasma. Furthermore, in scalded mice models combined with SSTIs induced by either Methicillin-Resistant *Staphylococcus aureus* (*MRSA*) or *Candida albicans*, GK-19 showed significant antimicrobial and healing effects. Overall, it was demonstrated that GK-19 might be a promising drug candidate in the battle against drug-resistant bacterial and fungal infections.

## 1. Introduction

The misuse and overuse of antimicrobial drugs in the clinic is very common in recent years [1,2,3,4]. The drug resistance of the top five pathogenic bacteria in nosocomial infection continues to deteriorate [1,5]. Some drug-resistant bacteria and even superbugs are no longer rare in the clinic [6,7]. Moreover, the research cost of new antibiotics is relatively high, and the development cycle is much longer than the breeding speed of drug-resistant microbes. The number of new antibiotics approved every year is declining [8]. Therefore, in response to the rising challenge of drug resistance, it is particularly urgent to look for alternative antibiotics that will not induce drug-resistance [9,10].

Antimicrobial peptides (AMPs), a type of active peptides produced by the organism, are an important class of host-defense factors [11]. Compared with traditional antibiotics, it has been proven that antimicrobial peptides not only have broad-spectrum antibacterial properties, but also have significant properties such as antifungal, immune regulatory, antiviral, antiparasitic and even anti-tumoral [12,13,14]. More importantly, most AMPs are thought to kill bacteria cells by a mechanism of inducing membrane damage with subsequent leakage of bacterial cellular content/debris, which will not induce drug resistance, making them attractive scaffolds for the creation of therapeutics that are superior to traditional antibiotics [15,16,17]. Thus, it is expected to be a powerful weapon against infections, especially in the resistance to micro-organism infections.

The venom of scorpions, which has been proven to kill Gram-negative and Gram-positive bacteria, fungi, viruses and even tumor cells, are a rich source of antimicrobial peptides. *Androctonus amoreuxi* Antimicrobial Peptide 1 (AamAP1) [18], identified from the venom-derived cDNA library of the North African scorpion, *Androctonus amoeruxi*, is a new type of host defense peptide. With broad-spectrum antimicrobial properties, it has been posited that AamAP1 kills bacteria cells through membrane disruption, which will not induce bacterial resistance. However, there are still some disadvantages in using AamAP1. It displays moderate activity against Gram-positive and Gram-negative bacteria, however, AamAP1 and its derivatives (A3 and AamAP1-Lysine) are unstable in serum and have a predicted short half-life of less than two hours [19,20,21]. In addition, natural AamAP1 is difficult to obtain, and its purity is very low. More importantly, it is proved to be highly hemolytic and displays significantly high toxicity against mammalian cells. All these disadvantages have greatly limited its clinical application prospects.

In this study, the amino acid sequence of a new scorpion venom antimicrobial peptide analogue GK-19, derived from AamAP1 and its derivatives, was designed and evaluated. The antimicrobial activity and mechanism of GK-19 for both bacteria and fungi were investigated. At the same time, the therapeutic effect of GK-19 on scalded mice models combined with skin and soft tissue infections (SSTIs) induced by Methicillin-Resistant *Staphylococcus aureus* (*MRSA*) or *Candida albicans* (*C. albicans*) was studied to determine its applicability. The results demonstrated that GK-19 has broad-spectrum antimicrobial activities for both bacteria and fungi by disrupting the microbial cell membrane. Cellular and in vivo studies proved that GK-19 showed negligible toxicity to mammalian cells, low hemolytic activity and high stability in plasma. Furthermore, in scalded mice models combined with skin and soft tissue infections induced by either *MRSA* or *C. albicans*, GK-19 showed significant antimicrobial and healing effects. Thus, the novel scorpion venom-derived peptide analogue GK-19 is a promising drug candidate in the battle against multi-resistant bacterial and fungal infections.

## 2. Materials and Methods

### 2.1. Materials

All the antimicrobial peptides were purchased from GL Biochem (Shanghai) Ltd. (Shanghai, China) and their purity was determined to be higher than 98% using reversed-phase high-performance liquid chromatography (RP-HPLC) and mass spectrometry. Mueller Hinton Broth (MHB), Mueller Hinton Agar (MHA), Potato Dextrose Broth (PDB), Potato Dextrose Agar (PDA), clindamycin hydrochloride (CDHC), and fluconazole were purchased from Solarbio Life Science (Beijing, China). CCK-8 kit was obtained from Dojindo Molecular Technologies, Inc. (Tokyo, Japan). Urea assay kit, alanine aminotransferase assay kit, aspartate aminotransferase assay kit and creatinine assay kit were purchased from Nanjing Jiancheng Bioengineering Institute (Nanjing, China). Acetonitrile (ACN) and trifluoroacetic acid (TFA) were purchased from Thermo Fisher Scientific (Waltham, MA, USA). SYPRO Orange protein gel stain was purchased from Sigma-Aldrich (St. Louis, MO, USA). All the reagents were of commercial special grade and were used without further purification.

### 2.2. Characterization of the Physicochemical Properties of the Antimicrobial Peptides

The physicochemical properties of the designed antimicrobial peptides were calculated using the peptide property calculating tools including SOPMA secondary structure prediction method, Expasy ProtParam tool and Innovagen Proteomics tools based on the peptide sequence. Thermal shift assay was conducted on the Bio-Rad CFX Connect^TM^ Real-Time system using 5× SYPRO Orange dye, while the concentration of antimicrobial peptides was 10 μM. The heating procedure was 10 °C to 95 °C with a heating rate of 1.5 °C/min. Data analysis was processed using the CFX Maestro software (4.1.2433.1219, Hercules, CA, USA).

### 2.3. Antimicrobial Peptides Stability Assay

Antimicrobial peptides were dissolved in water or rat serum with a concentration of 10 mg/mL. The solution was incubated at 37 °C, 200 rpm for different time periods, and diluted 50 times for the HPLC analysis. In water, the incubation time was 0 h and 48 h. In rat serum, the incubation time was 0, 2, 4, 7, 12, 24 or 48 h. Each incubation condition was repeated three times. HPLC was conducted on a Waters HPLC system, which consisted of a 2996 photodiode array detector, a 2695 separation module and a temperature control column oven. Column: Sinochrom ODS-BP, 5 μm, 4.6 × 250 mm (Dalian Elite Analytical Instruments Co., Ltd., Dalian, China). Mobile phase: A: 100% ACN + 0.1% TFA; B: 100% Water + 0.1% TFA. Gradient elution: 0–20 min, 95% B–100% A; 20–25 min, 100% A. Detection wavelength: 220 nm. Flow rate: 1 mL/min. Sample volume: 50 μL (0.2 mg/mL).

### 2.4. Bacteria and Fungi Strains Preparation and Growth Conditions

*Pseudomonas aeruginosa* (*P. aeruginosa*, ATCC27853), Methicillin-Resistant *Staphylococcus aureus* (MRSA, 2104270609, clinically isolated strain), *Escherichia coli* (*E. coli*, ATCC25922), *Klebsiella pneumoniae* (*K. pneumoniae*, ATCC700603), *Enterococcus faecalis* (*E. faecalis*, ATCC29212), *Candida krusei* (*C. krusei*, ATCC6258), *Candida albicans* (*C. albicans*, ATCC90028), and *Candida glabrata* (*C. glabrata*, ATCCMYA-2950) were obtained from the First Affiliated Hospital of Xi’an Jiaotong University. Bacterial strains were cultured in MHA or MHB. Fungal strains were cultured in PDA or PDB.

### 2.5. Antimicrobial Assay

Strains were incubated in MHB or PDB. After growing for 12–16 h at 37 °C, 200 rpm, 100 μL of 1 × 10^6^ CFU/mL of the strains was seeded into 96-well plates and incubated with fresh culture medium containing various antimicrobial peptides (peptide concentration: 0, 0.5, 1.0, 2.0, 3.0, 5.0, 10, 15 or 20 μM) for 12 h. Each concentration was repeated five times. Absorbance at 600 nm was measured using the Multilabel Reader Varioskan Flash (Thermo Fisher). Microbial viability was calculated using the following equation:

Viability (%) = (mean absorbance value of treatment group − mean absorbance value of blank group)/(mean absorbance value of control group − mean absorbance value of blank group) × 100. (1)

### 2.6. Antimicrobial Mechanism

Scanning electron microscopy (SEM, GeminiSEM 500, Carl Zeiss, Oberkochen, Germany) was used to investigate the antimicrobial mechanism of the designed peptides as reported in references [17,22]. Different strains were incubated with 50 μM of GK-19 for 2 h at 37 °C, 200 rpm. The solution was centrifuged at 4000 rpm for 10 min and washed with PBS twice. The supernatant was removed and 2.5% of glutaraldehyde solution was added for fixation of the microbial clumps. After a standard SEM sample preparation process, the samples were placed on carbon tape adhered to an aluminum stud and coated with gold before SEM analysis.

### 2.7. Cells and Animals

Rat pulmonary artery smooth muscle cells (RPASMCs, primary cells separated by our laboratory) and human keratinocytes cells (HaCaT cells, Mingjing Biology, Shanghai, China) were cultured in high-glucose Dulbecco’s Modified Eagle Medium (DMEM), supplemented with 10% fetal bovine serum (FBS). Human umbilical vein endothelial cells (HUVECs, BeNa Culture Collection, Kunshan City, China) were cultured in the MCDB131 medium supplemented with 20% FBS and growth factor additives. Human embryonic kidney cells (293, Procell Life Science & Technology Co., Ltd., Wuhan, China) were cultured in minimum Eagle’s medium (MEM) supplemented with 10% FBS. All cells were cultured at 37 °C, with 5% CO_2_. All animals used in this study were supplied by the Medical Animal Test Center of Xi’an Jiaotong University and study protocols were approved by the Institutional Animal Care and Use Committee of the Ethics Committee of Health Science Center, Xi’an Jiaotong University (Ethical approval code: 2017-609).

### 2.8. In Vitro Cytotoxicity Assay

Cells were inoculated in 96-well plates at 5 × 10^3^ cells/well and cultured at 37 °C under 5% CO_2_. The supernatant was removed, and the cells were washed three times with PBS. Next, 100 μL of fresh culture medium containing different antimicrobial peptides was added (peptide concentration: 0, 1.0, 2.0, 5.0, 10, 25, 50 and 100 μM). Each concentration was repeated five times. Then, 10 μL of CCK-8 reagent was added 48 h later, followed by another two hours of incubation. The absorbance at 450 nm was measured using the same Multilabel Reader. Cellular growth viability was calculated using the following equation:

Viability (%) = (mean absorbance value of treatment group − mean absorbance value of blank group group)/(mean absorbance value of control group − mean absorbance value of blank group) × 100. (2)

### 2.9. In Vivo Cytotoxicity Assay

Forty healthy male Kunming mice weighing ~25 g (5–6 weeks old) were injected intravenously with 100 μL of GK-19 with concentrations of 5 μmol/kg (n = 15) or 25 μmol/kg (n = 15). Mice blood samples (n = 5, per group and per time point) were collected from the mice’s eyeballs on days 1, 3 and 7 after injection for renal function test and liver function test. Meanwhile, major organs including lungs, heart, kidneys, liver and spleen were also harvested for hematoxylin and eosin staining on days 1, 3 and 7 after injection. To monitor the body weight changes in mice after injection of GK-19, five healthy male Kunming mice weighing ~25 g were injected intravenously with 100 μL of GK-19 with concentrations of 25 μmol/kg. Mice weights were recorded daily for seven days.

### 2.10. Hemolysis Assay

The hemolysis assay was carried out as reported in the literature [22]. Briefly, healthy male rats weighing ~300 g were anesthetized by isoflurane using a small animal anesthesia machine (R640, RWD Life Science, Shenzhen, China). Fresh rat blood samples were drawn from the ventral main vein and collected into an ethylene diamine tetraacetic acid (EDTA) treated anticoagulation tube. The collected blood samples were centrifuged at 4 °C, 350× *g* for five minutes. Red blood cells (RBCs) were pipetted and washed three times with cold PBS. Then, 20 μL of RBC suspension in PBS (8% in volume) was mixed with 20 μL of GK-19 (GK-19 concentrations: 5, 25, 50, 100, 200 and 400 μM), PBS, or 1% Triton X-100 in 1.5 mL Eppendorf (EP) tubes. These tubes were incubated at 37 °C, 1000 rpm in a BG200 dry bath incubator (Hangzhou LongGene Scientific Instruments Co., Ltd., Hangzhou, China) for 1 h. Then, the tubes were centrifuged at 350× *g* for 5 min. Next, 20 μL of the supernatants was mixed with 180 μL of PBS. Afterwards, 100 μL of the mixture was transferred into 96-well plates and hemoglobin release was monitored at 576 nm using a Multilabel Reader Varioskan Flash (Thermo Fisher). The RBC suspension in PBS was used as a negative control. The absorbance of the wells containing 1% Triton X-100 was taken as 100% hemolysis. The percentage of hemolysis was calculated using this formula:

Hemolysis (%) = [(OD_576 nm_ of the peptide solution − OD_576 nm_ of the negative control)/(OD_576 nm_ of the positive control − OD_576 nm_ of the negative control)] × 100. (3)

The tests were repeated six times, and the data were expressed by the mean ± standard deviation of the six replicates.

### 2.11. The Scalded Mice Models Combined with SSTIs

The scalded mice models combined with SSTIs by *MRSA* or *C. albicans* were constructed [23]. Fifty normal male Kunming mice (5–6 weeks old) weighing ~25 g were anesthetized by isoflurane using a small animal anesthesia machine (R640, RWD Life Science, China). After skin preparation and disinfection, a custom-made circular iron (diameter: 8 mm) (shown in Figure S4) was applied to both sides of the spine of the mice for 10 s at 300 °C, resulting in four scalded wounds. Mice were injected subcutaneously with 5 mg/kg meloxicam (Aladdin, Shanghai, China) for four days (once per day) after scalding for pain relief. Two days after scalding, the skin tissues of the scalded areas were removed carefully, and about 30 μL of 1 × 10^7^ CFU/mL of *MRSA* or *C. albicans* was applied evenly onto the surfaces of the wounds. The mice were returned to the original feeding environment after the microbial fluid was completely absorbed (1.5–2 h), with two mice per cage. Corncob granules with SPF level were used as padding.

### 2.12. In Vivo Anti-SSTIs Assay

Two days after microbe inoculating, the scalded wounds of each mouse were divided into four groups randomly. Saline solution, antimicrobial peptide, clindamycin hydrochloride (for *MRSA*), or fluconazole (for *C. albicans*) were applied evenly onto the surfaces of the wounds twice a day (morning and evening) and the drug concentration was kept at 50 μM. The mice were returned to the original feeding environment after the solutions were completely absorbed. The treatment lasted for 12–16 days. Photographs of the wound areas were taken every two days using an iPhone 11 and the wound areas were measured every two days using Image J software (152-win-java8, Bethesda, MD, USA). The average wound areas on day two were used as the reference (100% unhealed) to normalized wound areas. Skin tissues of the wound areas were removed carefully at different time points (n = 5 for each time point) and weighted in a sterile environment. For *MRSA* infection, 10 mg of the wound tissues was dispersed into 1000 μL of sterile saline solution and the tissue solution was ground into suspension in a tissuelyser (Tissuelyser LT, Servicebio). Then, the tissue suspension was diluted with sterile saline solution 10 times and plated on MHA with a sample volume of 50 μL. For *C. albicans* infection, 10 mg of the wound tissues was dispersed into 200 μL of sterile saline solution and the tissue solution was also ground into suspension in the tissuelyser. The tissue suspension was then plated on PDA directly with a sample volume of 20 μL. After culturing overnight, the colonies were counted using Image J software or human eyes. In addition, the skin tissues of the wound area were removed carefully at different time points and dispersed into a 4% paraformaldehyde solution for further use.

### 2.13. Histological Analysis

The hematoxylin and eosin staining in this study was carried out by Servicebio (Wuhan, China). Briefly, the obtained skin tissues were soaked in 4% paraformaldehyde solution (Beyotime, China) and fixed for more than 24 h. Then, they were dehydrated and embedded with paraffin, and tissue sections having thicknesses of 4 μm were prepared. After deparaffinization and rehydration, hematoxylin and eosin staining was performed. Dehydration and mounting were then performed, and sections were observed with a Leica slide scanning microscopic imaging system (SCN400).

### 2.14. Statistical Analysis

The data were expressed as means ± standard deviation (SD). The statistical difference between the two groups was determined using Student’s *t*-test (GraphPad Prism 8.0.1.244, San Diego, CA, USA) with the following *p*-values: * *p* < 0.05, ** *p* < 0.01, *** *p* < 0.001.

## 3. Results

### 3.1. Peptide Functional Screening Showed a Prolonged Half-Life of GK-19

Based on the amino acid sequence of AamAP1, its derivatives A3 and AamAP1-Lysine, a glycine residue was introduced at the N-terminal end of AamAP1-Lysine. As shown in Table 1, with the introduction of glycine, the hydrophobicity of GK-19 decreased slightly and the helicity increased. More importantly, the predicted half-life was found to be greatly extended, suggesting that its antimicrobial activity may also be enhanced. The melt curve and melt peak in Figure S1 showed that the changes in amino acid sequence and numbers did not affect the thermal stability of peptides. Subsequently, the stability of AamAP1 and GK-19 in water and rat serum was investigated by HPLC. When preserved in water, both GK-19 and AamAP1 retained their structural stability for 48 h or more, even at the physiological temperature of 37 °C (Figure S2a). However, the structure of AamAP1 changed and the position of its chromatographic peak moved backward when incubated in rat serum for only two hours. More than 65% of AamAP1 peptides was degraded after seven hours of co-incubation with rat serum. In contrast, GK-19 maintained its stability even after 24 h of co-incubation with rat serum and more than 50% of GK-19 still remained (Figure S2b,c). These results encouraged further study of the antimicrobial activity of GK-19.

### 3.2. GK-19 Potently Kills a Broad Range of Both Bacteria and Fungi by Permeabilizing the Microbial Membrane

**Figure 1:**
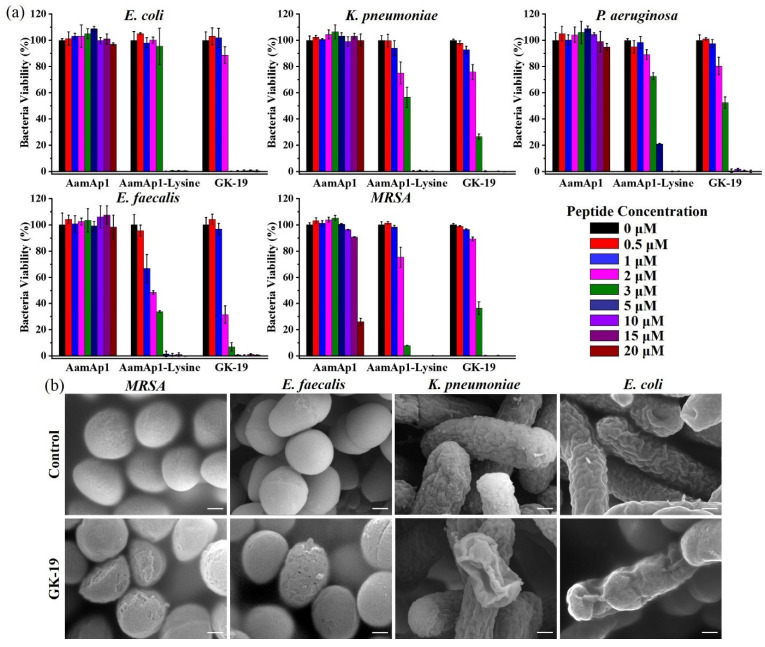
Antibacterial activity of AamAP1, AamAP1-Lys and GK-19. (**a**) Dose-dependent survival of *E. coli*, *K. pneumoniae*, *P. aeruginosa*, *E. faecalis* and *MRSA* treated with different antimicrobial peptides for 12 h. Bacteria (1 × 10^6^ CFU/mL) were incubated with the peptides at concentrations varying from 0 to 20 μM. (n = 5) (**b**) SEM images of different bacteria after 2 h of incubation with GK-19 at a concentration of 50 μM. Scale bar: 200 nm.

As shown in Figure 1a and Table S1, GK-19 exhibited potent antibacterial activity against three Gram-positive and two Gram-negative bacteria with much lower minimal inhibitory concentration (MIC) values ranging between 3 and 10 μM (*E. coli* (3 μM), *K. pneumoniae* (5 μM), *P. aeruginosa* (5 μM), *E. faecalis* (3 μM), and *MRSA* (5 μM)) in comparison to that of AamAP1 (>20 μM). In contrast, Aamap1 had almost no bactericidal effect within the same concentration range. Additionally, except for *MRSA*, GK-19 had stronger antimicrobial activity against the other four common clinical pathogenic bacteria than AamAP1-Lysine. To assess the possible mechanism of GK-19′s action on bacteria, two Gram-positive bacteria (*E. faecalis* and *MRSA*) and two Gram-negative bacteria (*E. coli* and *K. pneumoniae*) were incubated with GK-19 at a concentration of 50 μM for 2 h. As illustrated in Figure 1b, GK-19 led to the formation of blebs (roughness) and irregularly shaped holes on the bacterial membrane of both *E. faecalis* and *MRSA*, whereas untreated controlled groups revealed a significant difference in membrane morphology with smooth and complete bacterial membrane. For Gram-negative bacteria, *E. coli* and *K. pneumoniae*, after treatment with GK-19, irregular abysses, pores, and ruptures were also observed. Considering the known mechanisms of the antibacterial peptides, it is conceivable that disruption of the bacterial membrane and/or cell wall structure is the cause of lethality in bacteria treatment with GK-19.

**Figure 2:**
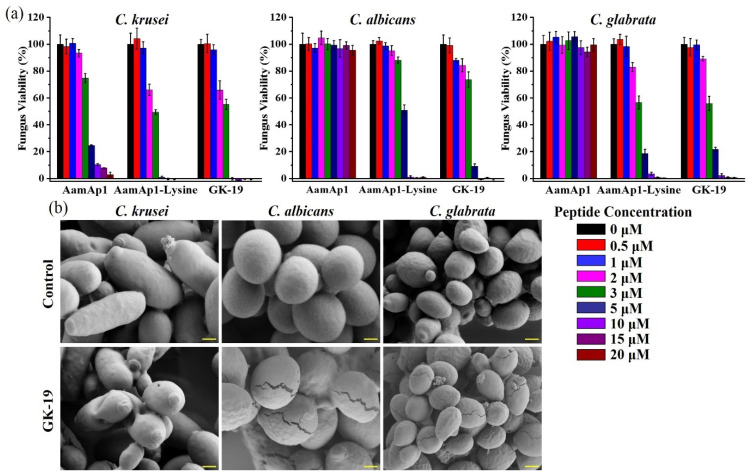
Antifungal activity of AamAP1, AamAP1-Lys and GK-19. (**a**) Dose-dependent survival of *C. krusei*, *C. albicans* and *C. glabrata* treated with different antimicrobial peptides for 12 h. Fungi (1 × 10^6^ CFU/mL) were incubated with the peptides at concentrations varying from 0 to 20 μM. (n = 5) (**b**) SEM images of different fungi after 2 h of incubation with GK-19 at a concentration of 50 μM. Scale bar: 1 μm.

Encouraged by the excellent antibacterial activity of GK-19, three common *Candida* species were then used to investigate the antifungal activity of GK-19. As shown in Figure 2a and Table S1, GK-19 also exhibited distinguished antifungal activity with much lower minimal inhibitory concentration (MIC) values ranging between 5 to 10 μM (*C. krusei* (5 μM), *C. albicans* (10 μM), and *C. glabrata* (10 μM)) in comparison to that of AamAP1. AamAp1 could only inhibit 90% of *C. krusei* and showed almost no fungicidal effect on *C. albicans* and *C. glabrata* within the same concentration range. In addition, AamApl-Lysine showed similar antifungal activity as GK-19. These results suggested that the antimicrobial activity of GK-19 against these microorganisms was maintained or even enhanced by introducing a glycine residue to the N-terminus of AamAP1-Lysine. Meanwhile, the antifungal mechanism of GK-19 by using the same method as bacteria was also studied. As illustrated in Figure 2b, after treatment with GK-19, the cells of *C. krusei* also showed membrane disruption and pore formation in most cells in comparison with untreated group. Deep cracks and ruptures appeared on the membranes of *C. albicans* and *C. glabrata,* slightly different from the membrane morphology of *C. krusei*. It is conceivable that the disruption of the fungal membrane and/or cell wall structure was also the cause of lethality in fungi treatment with GK-19. Collectively, these data suggested that GK-19 exhibits potent and quick antibacterial and antifungal activities by permeabilizing the microbial membrane, indicating that it might be used for the treatment of infections both in vitro and in vivo without inducing resistance.

### 3.3. GK-19 Showed Negligible Toxicity Both In Vitro and In Vivo

**Figure 3:**
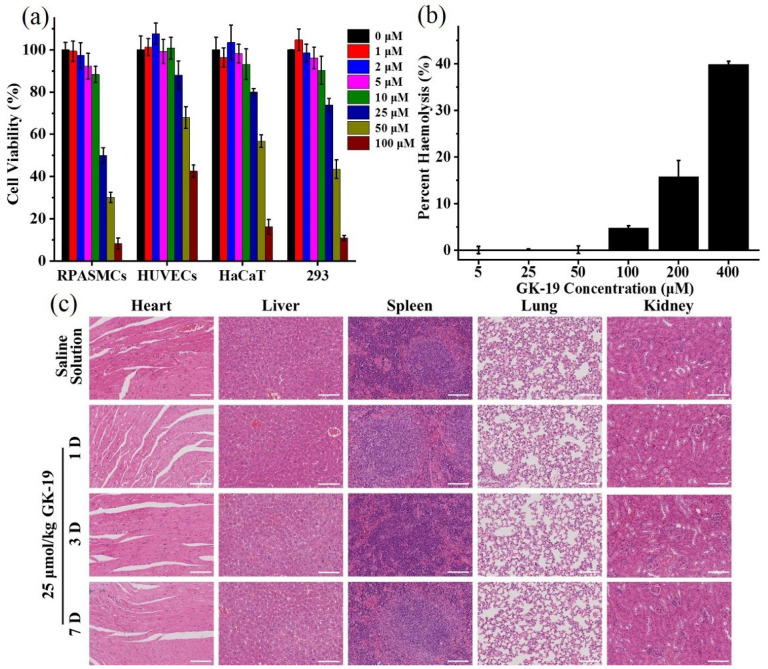
(**a**) Toxicity of GK-19 to mammalian cells. RPASMCs, HUVECs, HaCaT and 293 cells were cultured in mediums containing different concentrations of GK-19 (0–100 μM) for 48 h. (n = 5). GK-19 caused a negligible toxicity to mammalian cells at effective antimicrobial concentrations. (**b**) Hemolytic activity of GK-19 on rat erythrocytes. (**c**) Histology analysis of various organs of the mice treated with saline solution or GK-19 (25 μmol/kg), respectively. The heart, liver, spleen, lungs and kidneys were harvested on days 1, 3 and 7. No observable tissue damage, necrosis or inflammation were observed. Scale bar: 200 μm.

To better understand the toxicity of the GK-19, several normal mammalian cells, including rat pulmonary artery smooth muscle cells (RPASMCs), human keratinocytes cells (HaCaT), human umbilical vein endothelial cells (HUVECs), and human embryonic kidney cells (293), were chosen to evaluate the cytotoxicity induced by GK-19. As shown in Figure 3a, almost no apoptotic cells were observed in the presence of GK-19 at the effective antimicrobial concentration (10 μM). At 10 times the effective antimicrobial concentration (100 μM), treatment with GK-19 resulted in cell viability of less than 20% for RPASMCs, HUVECs, and 293 cells. However, more than 42% of the HaCaT cells still survived after treatment with 100 μM of GK-19. In addition, the hemolytic activity of GK-19 in vitro was evaluated. As shown in Figure 3b, GK-19 exhibited negligible hemolytic activity even at relatively high concentrations (100 μM). To further evaluate the safety of GK-19 in vivo, the mice were injected intravenously with GK-19 at the dosage of 5 μmol/kg or 25 μmol/kg. As shown in Figure 3c, the slices of mice’s major organs treated with 25 μmol/kg of GK-19 showed no observable tissue damage, necrosis, or inflammation, either in the short term of 1 day or the long term of seven days. Meanwhile, the results of liver and renal function tests showed no difference from that in healthy mice (Figure S3a–d). The changes in the body weight of the mice were also monitored for seven days. As shown in Figure S3e, the mice remained healthy as indicated by the gradual gain in body weight, which further demonstrated that the GK-19 was relatively safe for use in vivo. Collectively, these data confirmed a favorable toxicity/safety profile of GK-19 in vitro and in vivo.

### 3.4. GK-19 Promoted the Wound Healing of Mice against SSTIs Caused by MRSA

**Figure 4:**
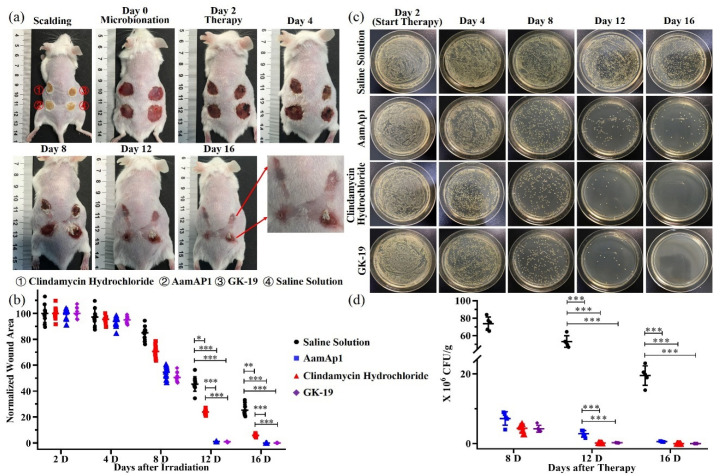
The bactericidal and healing effect of GK-19 on *MRSA* inoculated onto scalded dorsal skin of mice. (**a**) Time course images of mice infected by *MRSA*. Infected dorsal skins of the mice were treated with saline solution, AamAp1, clindamycin hydrochloride, or GK-19, respectively, twice every day (n = 10). The concentrations of drugs was 50 μM. (**b**) Quantitative analyses of wound areas using Image J software. (**c**,**d**) Time course bacterial count in *MRSA* infected wounds after treatment with different antimicrobial peptides. Skin tissues of different groups were sampled on days 2, 4, 8, 12 and 16. Tissue suspensions were diluted with sterile saline solution and 50 μL of tissue suspensions was then plated in the MHA culture plates. Images were taken 18–24 h after inoculation. The quantitative analyses of colonies were performed using Image J software or visual observation (n = 5). * *p* < 0.05, ** *p* < 0.01, *** *p* < 0.001.

**Figure 5:**
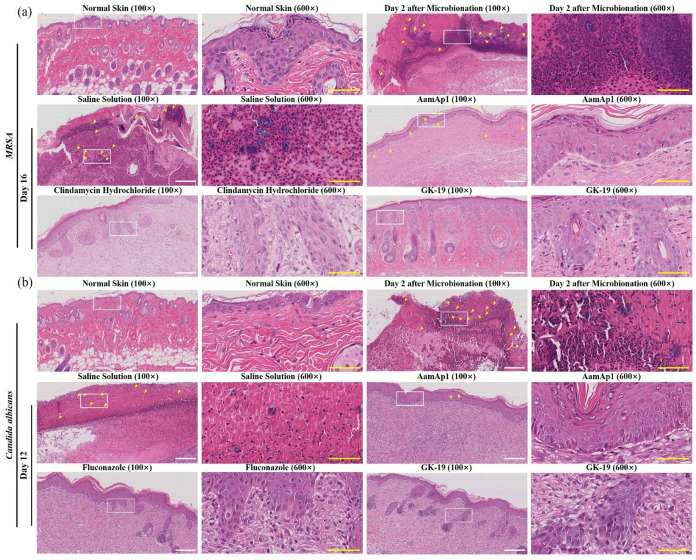
The histology analysis of skin samples of the scalded mice infected by *MRSA* (**a**) or *C. albicans* (**b**). The skin wounds were treated with saline solution, AamAP1, GK-19, clindamycin hydrochloride (for *MRSA* infection), or fluconazole (for *C. albicans* infection), respectively, and the concentration of drugs was 50 μM. Skin samples were harvested at different time points. White scale bar: 200 μm. Yellow scale bar: 50 μm. Yellow arrows indicate the location of bacterial or fungal clumps.

Encouraged by the promising in vitro and in vivo results discussed above, the in vivo antimicrobial efficacy of GK-19 using scalded mice models combined with SSTIs by *MRSA* was further assessed. Figure 4a,b shows the gross appearances of the skin wounds and quantitative analysis of the wound area. After scalding, the skin of the scalded area became slightly focal yellow and hard immediately. The wounds were treated with saline solution (SS), AamAp1, clindamycin hydrochloride (CDHC), or GK-19, two days later, respectively, and observed until they healed. On day two, the skin wounds of the mice were dark brown and edematous. Scabs were tightly adherent to the wound surface on day four in GK-19 or CDHC treated groups, whereas the skin tissue edema was aggravated and some pus was observed in AamAp1 and SS treated groups. On day eight, the sizes of the scabs treated by GK-19 or CDHC were significantly reduced with more than 45% of the wound area healed in comparison with those treated by AamAp1 (~27%) or SS (~13%). On day 12, the scab tissues of GK-19 or CDHC treated wounds fell off and wound areas were greatly reduced. In contrast, scabs still tightly adhered to the tissues treated by AamAp1 or SS. However, the wound healing rate of the AamAP1 treated group (~77%) was found to be significantly faster than that of the SS treated group (~54%), indicating that AamAP1 also had a certain ability to promote wound healing. The wounds in both GK-19 and CDHC treated groups had completely disappeared on day 16, whereas ~6% or ~27% of unhealed wounds remained observable in AamAp1 or SS treated groups, respectively. These results suggested that Gk-19 was comparable to CDHC and more effective than AamAP1 in promoting wound healing of SSTIs caused by *MRSA*. Meanwhile, the colonized *MRSA* in the skin and subcutaneous tissues was quantified. As shown in Figure 4c,d, compared with SS groups, treatment with GK-19 led to a significant reduction of *MRSA*, with more than 95% of *MRSA* being suppressed at day eight, which was comparable to the inhibition rate of CDHC (~95%). However, AamAP1 groups also showed a *MRSA* inhibition rate of more than 90%, but a much lower healing speed. This could be attributed to the serious inflammatory reaction caused by the existing abundant *MRSA* at the early period of infection. To further evaluate the antibacterial and healing effect of GK-19 on infected wounds, histological analysis was performed. As shown in Figure 5a, compared with normal skin tissues, ubiquitous inflammatory cell infiltration (mainly neutrophil), large amounts of fragments of pus cells, necrotic tissues, and *MRSA* clumps were observed in the skin tissues two days after infection by *MRSA*. On day 16, the healing effect of GK-19 was comparable to that of CDHC, with completely healed wound tissues. No *MRSA* clumps or pus cell fragments were observed, and inflammatory cells were less distributed or not observed. Moreover, a large number of fibroblasts, significant neovascularization and even newborn hair follicle tissues were observed in the subcutaneous tissue just below the wound surface of GK-19 treated groups, indicating good healing. In contrast, massive neutrophil infiltration and many smaller *MRSA* clumps were still observed in SS treated groups. In addition, the skin tissues of AamAP1 treated groups were nearly healed, but fewer *MRSA* clumps could also be observed. These data suggested that GK-19 could penetrate through the skin into the infected sites to eradicate *MRSA*, indicating its potential as part of a class of antibiotics with significant therapeutic potential for external use.

### 3.5. GK-19 Promoted the Wound Healing of Mice against SSTIs Caused by C. albicans

**Figure 6:**
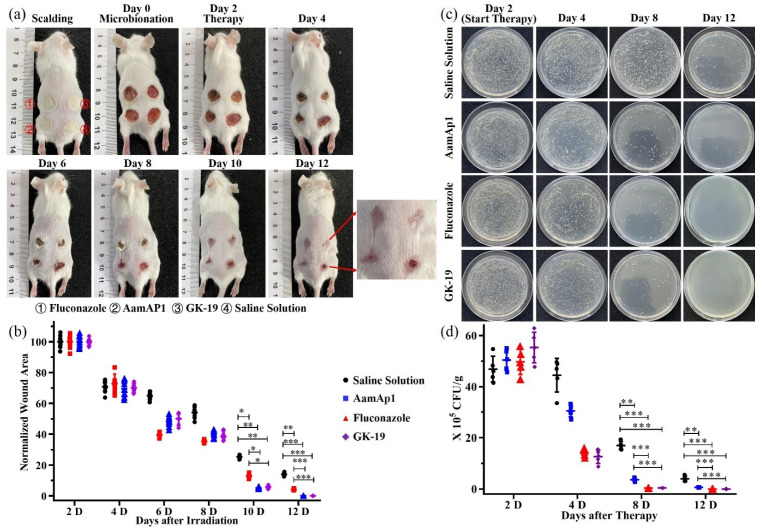
The fungicidal and healing effect of GK-19 on *C. albicans* inoculated onto scalded dorsal skin of mice. (**a**) Time course images of mice infected by *C. albicans*. The infected dorsal skin of mice was treated with saline solution, AamAp1, fluconazole, and GK-19, respectively, twice every day. The concentration of drugs was 50 μM. (**b**) Quantitative analyses of the wound areas using Image J software (n = 10). (**c**,**d**) Time course fungal count in *C. albicans* infected wounds after treatment with different antimicrobial peptides. Skin tissues of different groups were sampled on days 2, 4, 6, 8, 10 and 12. Tissue suspensions were diluted with sterile saline solution, and 20 μL of tissue suspensions was then plated in PDA culture plates. Images were taken 18–24 h after inoculation. The quantitative analyses of colonies were performed using Image J software or visual observation (n = 5). * *p* < 0.05, ** *p* < 0.01, *** *p* < 0.001.

Serious SSTIs are likely to develop into systemic infections that threaten the patient’s life and health [24]. Considering the growing threat of fungal infections and the excellent in vitro inhibitory effect on *Candida* of GK-19, scalded mice models combined with SSTIs by *C. albicans* were constructed as discussed above. The wounds were treated twice every day with saline solution (SS), AamAp1, fluconazole (FCZ), or GK-19, respectively, and observed until they healed. Similar results as in antibacterial studies were observed in Figure 6a,b. On day 10, the sizes of the scabs treated by GK-19 or FCZ were significantly reduced with more than 95% of the area of the wounds healed in comparison with those treated by AamAP1 (~88%) or SS (~75%). The wounds in both groups treated by GK-19 and FCZ had completely disappeared on day 12, whereas ~5% or ~14% of unhealed wounds remained observable in AamAp1 or SS treated groups. These results suggested that GK-19 was comparable to FCZ and more effective than AamAP1 in the aspect of promoting wound healing of SSTIs caused by *C. albicans*. Meanwhile, the colonized *C. albicans* in the infected skin and soft tissues were also quantified. As shown in Figure 6c,d, the fungal load in the tissues infected by *C. albicans* was much lower, which could be the reason for the faster healing speed of SSTIs induced by *C. albicans*. Compared with SS treated groups, treatment with GK-19 could lead to a significant reduction in the colonization of *C. albicans* in subcutaneous tissues, with more than 97% of *C. albicans* being suppressed at day eight, which was comparable to the inhibition rate of FCZ (~98%). In contrast, there were still more than 20% residual *C. albicans* in the group treated by AamAP1 in comparison with the SS treated group. On day 12, there were no *C. albicans* detected in groups treated by GK-19 or FCZ, whereas a few residual *C. albicans* were still observed in SS and AamAP1 treated groups. These results suggested that GK-19 indeed promoted wound healing by inhibiting fungal proliferation.

Histological analysis was also performed. As expected, the number of fungal clumps observed in the tissues infected by *C. albicans* was smaller and the clumps tended to be round or oval, which corresponded to the previous results of fungal load (Figure 5b). Ubiquitous inflammatory cell infiltration, monocytes, lymphocytes, and neutrophils were found to be infiltrated and necrotic tissues were observed in the skin tissues two days after infection by *C. albicans*. On day 12, the wounds of both GK-19 and FCZ treated groups were completely healed, with no observable *C. albicans* clumps or inflammatory cells. Moreover, the skin structure was almost completely intact with significant neovascularization and a large number of newborn hair follicle tissues, indicating the comparable healing effect of GK-19 to FCZ. In contrast, there was significant neutrophil infiltration, a large number of lymphocytes, a small number of necrotic tissues and many smaller *C. albicans* clumps observed in the SS treated groups, and wound tissues were still scabby. In addition, the skin tissues of AamAP1 treated groups were nearly healed, but a small number of very small *C. albicans* clumps could also be observed. These data suggested that GK-19 could also penetrate through the skin into the infected sites to eradicate *C. albicans*, indicating its potential as part of a class of antibiotics with significant therapeutic potential for SSTIs caused by both bacteria and fungi.

## 4. Discussion

As the cornerstones of modern public health, antibiotics have made remarkable contributions to anti-infection. However, the widespread use and even abuse of antibiotics have led to increasing resistance in many important human pathogens [25]. Meanwhile, the focus of anti-infections in the clinic is to fight bacteria for a long period, but fungal infections have been somewhat neglected. With the control of bacterial infections, fungal infections are gradually becoming an important disease [26]. Studies have shown that the prevalence of superficial mycosis worldwide is as high as 20–25%, indicating that fungi have partially replaced bacteria as the main pathogen [27]. In addition, the over-treatment of bacteria can also lead to the growth of fungi. Untreated aggravation of deep SSTIs can cause rapid necrosis of tissues and organs, or it can spread into the blood, resulting in fungal septicemia and sepsis, thus threatening the patient’s life [28,29]. Therefore, the situation of infections caused by fungi is increasingly serious. Unfortunately, most existing antimicrobials tend to kill only bacteria or fungi, not both. Hence, there is an urgent need to find new antibiotic substitutes that will not induce resistance and have antimicrobial effect on both bacteria and fungi. Unlike antibiotics, which inhibit microbial growth by blocking the synthesis of microbial macromolecules in various ways, such as inhibiting protein, cell wall, and nucleic acid synthesis, antimicrobial peptides are generally considered to kill microbes by forming pores in microbial cell membranes and causing leakage of intracellular substances [15,30]. This antimicrobial mechanism is not believed to induce antimicrobial resistance in microorganisms [31]. Therefore, antimicrobial peptides are considered ideal substitutes for antibiotics with excellent market development prospects. At present, more than 2500 kinds of antimicrobial peptides have been discovered [26]. Some are approved by the Food and Drug Administration for preclinical and clinical studies [27,28]. It is worth noting that in the past 40 years, fewer than 50 antimicrobial peptides have entered the clinical stage worldwide. Therefore, research on antimicrobial peptides is quite meaningful and promising.

**Scheme 1:**
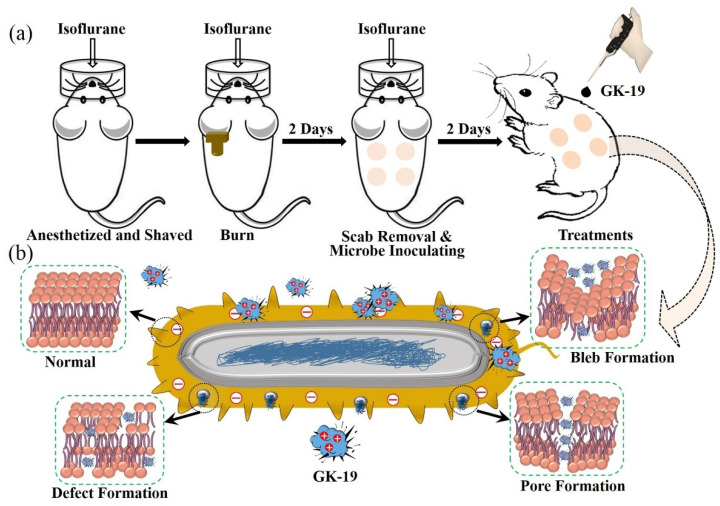
(**a**) Overall design of the animal experiment. (**b**) A schematic diagram of the possible mechanisms of antibacterial and antifungal properties of GK-19 in skin and soft tissue infections induced by *MRSA* or *Candida albicans*.

In this study, a novel 19-residue antimicrobial peptide called GK-19 was characterized. Synthetic GK-19 showed a wide range of antibacterial and antifungal activities. In particular, this study revealed that GK-19 can indeed induce the formation of pores by interacting with the cellular membranes of both bacteria and fungi, suggesting that GK-19 may not induce drug resistance (Scheme 1b). In addition, it was found that the morphologies of bacteria and fungi were quite different after incubation with GK-19. Formation of blebs (roughness) and irregularly shaped holes were observed in Gram-positive bacterial membranes of both *E. faecalis* and *MRSA*, irregular abysses and ruptures were observed in Gram-negative bacterial membranes of *E. coli* and *K. pneumonia*, and deep cracks and ruptures appeared on the membranes of *C. albicans* and *C. glabrata*, slightly different from the membrane morphology of *C. krusei*. This can be attributed to the different compositions of the cell membrane of Gram-positive bacteria, Gram-negative bacteria and fungi. The cellular membrane of Gram-positive bacteria consists of a lipid bilayer protected by a lipoteichoic acid layer, whereas the cellular membrane of Gram-negative bacteria is more complex because it consists of an inner and outer membrane separated by a periplasmic space, while the cellular membranes of fungi are different [29,32]. Therefore, the interactions between GK-19 and cellular membranes of different microorganisms are quite different. In addition, the interaction between GK-19 and the microbial cellular membrane appears to be the first step. It has been reported that AMPs can not only act on the cellular wall and cellular membrane but also enter the cell through direct penetration or endocytosis [33] and exert anti-microbial effects by targeting the nucleus, organelles, present in fungi, or intracellular proteins [34]. The cationic nature of GK-19 may also interfere with polyanionic intracellular components such as DNA, RNA and ribosomes etc. In addition, the transmembrane mechanisms of GK-19 are not fully understood. Therefore, further studies will be needed to determine the exact antimicrobial mechanism of GK-19 and its detailed interaction with bacteria and fungi.

The main obstacle in the clinical use of antimicrobial peptides as promising therapeutics is their side effects, such as cytotoxicity and hemolysis. The cellular membrane of bacteria is a target of cationic antimicrobial peptides due to the presence of electronegative lipids, while the structure of the human cell membrane is different from that of bacteria, so antimicrobial peptides can usually identify their target by this difference [35]. However, it has also been reported that mammalian cellular membranes can also be targeted by some antimicrobial peptides, meaning that antimicrobial peptides may bind to the host cell membrane and cause adverse reactions [36]. In addition, due to the broad-spectrum antibacterial and antifungal properties of antimicrobial peptides, the intrinsic beneficial flora in human bodies may also be influenced and killed, resulting in the disorder of beneficial flora in human bodies, and having serious impacts. Therefore, most of the FDA-approved antimicrobial peptide products are used topically or as rinse solutions. In this study, it was proved that GK-19 showed low toxicity to mammalian cells, so the first application direction chosen was also topical administration (Scheme 1a). GK-19 did show significant antimicrobial and healing effects on scalded mice models combined with SSTIs induced both by *MRSA* and by *C. albicans*. However, this is just a preliminary application study, and the method of administration was simple transdermal delivery. Meanwhile, this study only evaluated the therapeutic effect, but did not explore the molecular mechanism, which is a limitation of this study. In addition to acting directly on microorganisms, antimicrobial peptides can also promote the phagocytic activity of macrophages and the recruitment of lymphocytes, and affect the human immune system [33,37,38]. Antimicrobial peptides can even neutralize endotoxins produced by Gram-negative bacteria [39]. Therefore, further studies are needed to determine the molecular mechanism of GK-19 in promoting wound healing.

The stability of antimicrobial peptides was also found to be the main limitation of their use in vivo. Because of the quick digestion effect of peptidases and proteases in vivo and the neutralization of various ions in the blood, most of the antimicrobial peptides lost their antimicrobial activity in vivo. In this study, it was proved that GK-19 showed low hemolytic activity, high stability in plasma, and negligible damage to the functions of the liver and kidneys within the range of effective bactericidal and fungicidal concentrations, which provided a theoretical basis for administration of GK-19 through the tail vein. However, it was not investigated whether GK-19 would retain its superior antimicrobial activity after being injected into the body of mice by the tail vein, or whether it could be used for deep tissue infections, such as systemic infection, lung infection and other treatments [25]. There are still some challenges, and it should be noted that the influence associated with immediate and long-term administration with other GK-19 dosages is still unknown.

In addition, although GK-19 showed significant antimicrobial and healing effects in the treatment of SSTIs, the administration of daily and continuous dosing was used for SSTIs treated with GK-19, and this administration was quite cumbersome. Therefore, whether GK-19 can be combined with other technologies, such as hydrogels, to prepare antimicrobial skin dressing [40,41,42], from which peptide drugs can be released slowly for a long time, so as to reduce the frequency of administration and provide maximum convenience for patients, warrants further investigation. At the same time, the main reason affecting the application of existing antimicrobial peptides in deep tissue infections is that the antimicrobial peptides are cleared soon after entering the blood circulation system, resulting in a lower presence concentration in the lesion area than the effective concentration. The increase of the concentration of drug administration will enhance the toxicity and side effects on the body. For that reason, we are also considering whether we can try to combine GK-19 with nanotechnology [22,43,44]. We expect to find solutions to package GK-19 into nanomaterials for delivery, or directly modify GK-19 with nanomaterials. We expect that this combination will preserve the superior antibacterial properties of GK-19, enhancing its residing time in the bloodstream, increasing the binding time and presence of concentration in the lesion area, and increasing the antimicrobial effect with lower administration dosage.

## 5. Conclusions

To conclude, the results of this study demonstrated the potential of GK-19, a designed antimicrobial peptide analogue derived from scorpion venom, as a possible antimicrobial drug candidate which can not only inhibit Gram-positive and Gram-negative bacterial growth but also kill fungi by permeabilizing the microbial membrane. GK-19 showed low toxicity to mammalian cells, low hemolytic activity, high stability in plasma and significant antimicrobial and healing effects on scald combined with SSTIs induced by either *MRSA* or *C. albicans* in vivo. Thus, the novel scorpion venom-derived peptide analogue GK-19 is a promising drug candidate in the battle against multi-resistant bacterial and fungal infections.

## Figures and Tables

**Table 1:** Sequence and physicochemical properties of the designed peptides.

Peptide	AamAP1	AamAP1-Lysine	GK-19
Sequence	FLFSLIPHAIGGLISAFK	FLFKLIPKAIKKLISKFK	GFLFKLIPKAIKKLISKFK
Length	18	18	19
Hydrophobicity (H)	0.904	0.607	0.575
% Helicity	72.22	88.3	94.74
Net Charge z	+1.1	+6	+6
Polar Residues (n/%)	6/33.3	7/38.89	8/42.11
Nonpolar Residues (n/%)	12/66.67	11/61.11	11/57.89
Water Solubility	poor	good	good
Estimated Half-Life	1.1 h (mammalian reticulocytes, in vitro); 3 min (yeast, in vivo); 2 min (*Escherichia coli*, in vivo)		30 h; >20 h; >10 h

## Data Availability

The data presented in this study are available in the article and Supplementary Materials.

## Supplementary Materials

The following supporting information can be downloaded at: https://www.mdpi.com/article/10.3390/pharmaceutics14091937/s1, Figure S1: (a) Melt curve of AamAP1, AamAP1-Lys and GK-19. (b) Melt peak of AamAP1, AamAP1-Lys and GK-19; Figure S2: Stability of AamAP1 and GK-19 in water and rat serum. (a) The HPLC peaks of AamAP1 and GK-19 after incubation in water at 37 °C, 200 rpm for 0 and 48 h. (b,c) The HPLC peaks of AamAP1 and GK-19 after incubation in rat serum at 37 °C, 200 rpm for different times and quantitative analysis of undegraded peptides with peak area; Figure S3: In vivo toxicity of GK-19. Renal function test (a,b) and liver function test (c,d) of the mice treated with saline solution or GK-19 (5 and 25 μmol/kg). (e) Body weight changes in mice over 7 days after being injected with 25 μmol/kg of GK-19 (n = 5); Figure S4: Custom-made circular iron (diameter: 8 mm) for scalded mice models combined with SSTIs; Table S1: Minimum inhibitory concentration (MIC) of AamAP1, AamAP1-Lys and GK-19 against five bacteria and three fungi.

## Abbreviations

AamAP1: Androctonus Amoreuxi Antimicrobial Peptide 1; SSTIs: Skin and Soft Tissue Infections; AMPs: Antimicrobial peptides; RP-HPLC: reversed-phase high-performance liquid chromatography; MHB: Mueller-Hinton Broth; MHA: Mueller-Hinton Agar; PDB: Potato Dextrose Broth; PDA: Potato Dextrose Agar; ACN: Acetonitrile; TFA: trifluoroacetic acid; CCK-8: Cell Counting Kit-8; MRSA: Methicillin-Resistant Staphylococcus Aureus; *P. aeruginosa*: *Pseudomonas aeruginosa*; *E. coli*: *Escherichia coli*; *K. pneumoniae*: *Klebsiella pneumoniae*; *E. faecalis*: *Enterococcus faecalis*; *C. krusei*: *Candida krusei*; *C. albicans*: *Candida albicans*; *C. glabrata*: *Candida glabrata*; SEM: Scanning electron microscopy; RPASMCs: Rat pulmonary artery smooth muscle cells; HaCaT cells: Human keratinocytes cells; DMEM: high-glucose Dulbecco’s modified Eagle medium; FBS: fetal bovine serum; HUVECs: Human umbilical vein endothelial cells; 293: Human embryonic kidney cells; MEM: minimum Eagle’s medium.
