# Supplementary material for: Antibacterial and Antifungal Properties of a Novel Antimicrobial Peptide GK-19 and Its Application in Skin and Soft Tissue Infections Induced by MRSA or Candida albicans
Source: Pharmaceutics. 2022 Sep 13;14(9):1937. doi: 10.3390/pharmaceutics14091937 (PMC9503518; doi:10.3390/pharmaceutics14091937)
Supplement: Supplementary file 1 [file pharmaceutics-14-01937-s001.zip › pharmaceutics-1877334-supplementary.pdf]

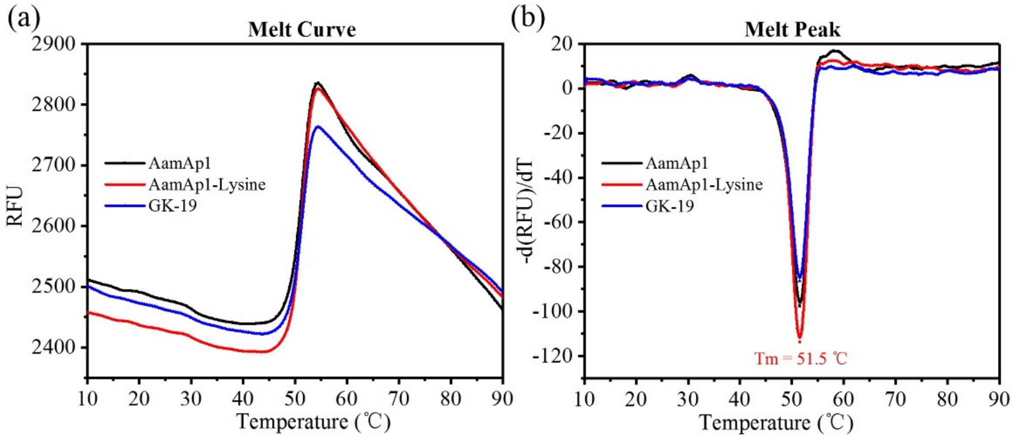

Figure S1 (**a**) Melt curve of AamAP1, Aamap1-Lys and GK-19. (**b**) Melt peak of AamAP1, Aamap1-Lys and GK-19.

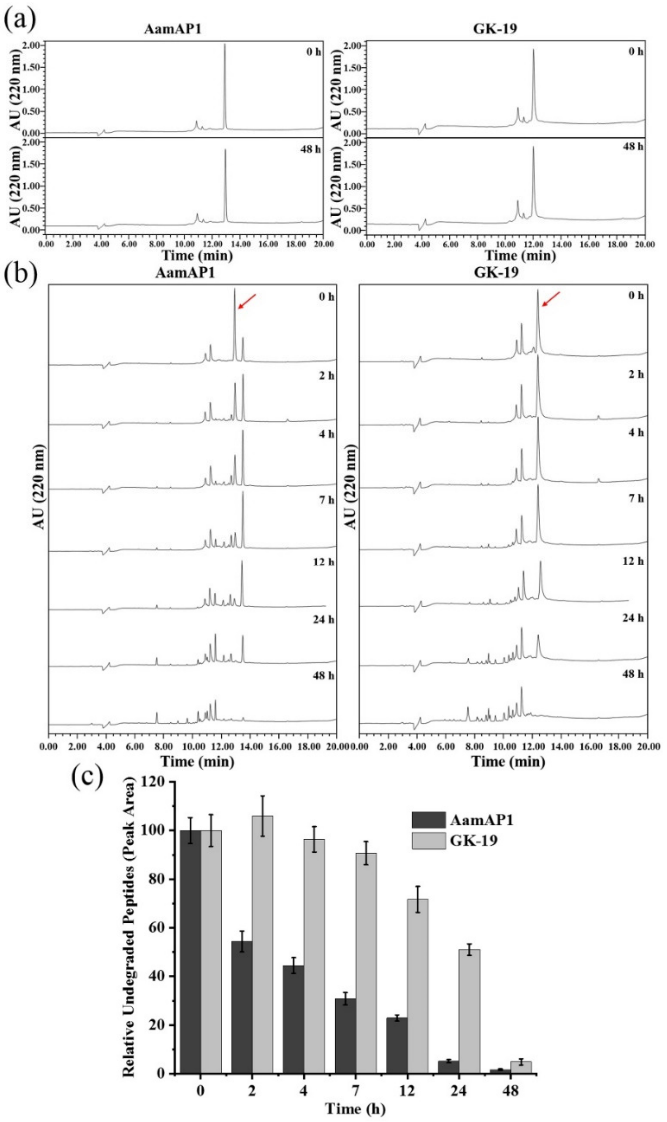

Figure S2 Stability of AamAP1 and GK-19 in water and rat serum. (**a**) The HPLC peaks of AamAP1 and GK-19 after incubation in water at 37 °C, 200 rpm for 0 and 48 h. (**b**,**c**) The HPLC peaks of AamAP1 and GK-19 after incubation in rat serum at 37 °C, 200 rpm for different times and quantitative analysis of undegraded peptides with peak area.

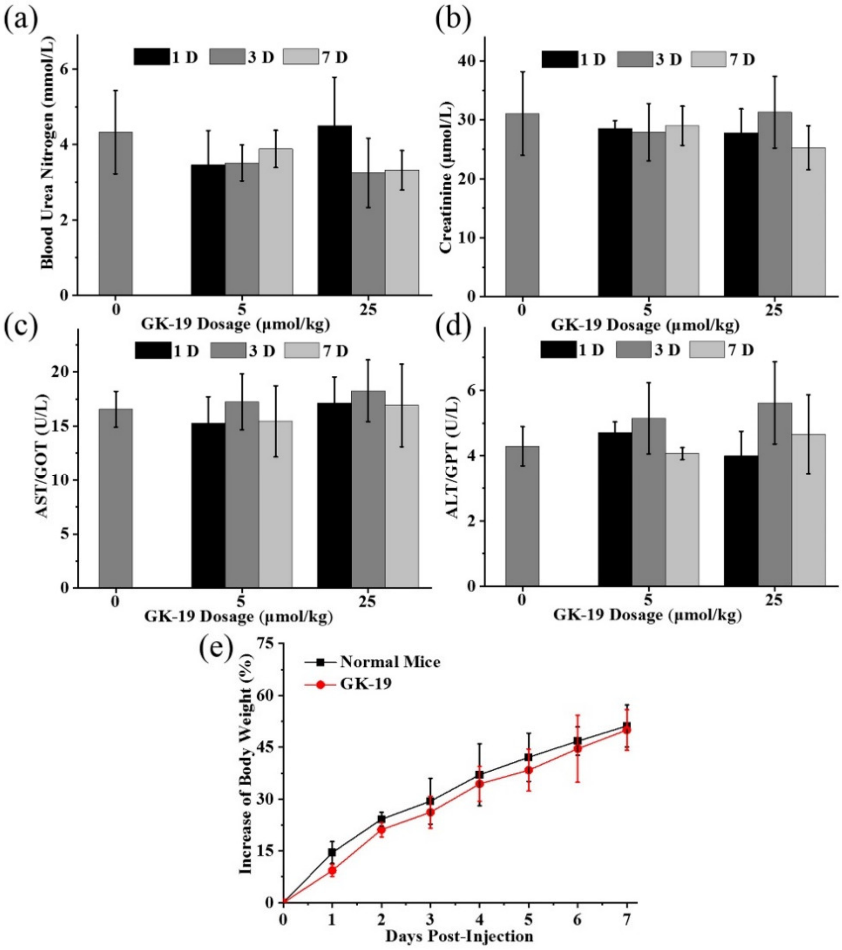

Figure S3 *In vivo* toxicity of GK-19. Renal function test (**a**,**b**) and liver function test (**c**,**d**) of the mice treated with saline solution or GK-19 (5 and 25 μmol/kg). (**e**) Body weight changes in mice over 7 days after being injected with 25 μmol/kg of GK-19 (n = 5).

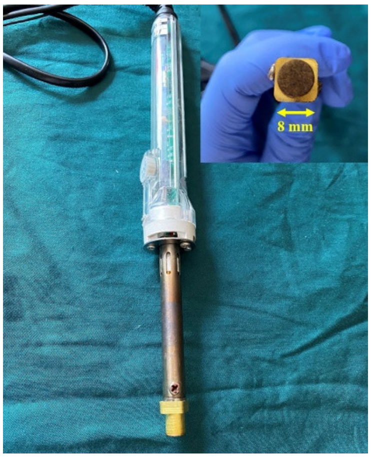

Figure S4 Custom-made circular iron (diameter: 8 mm) for scalded mouse models combined with SSTIs.

Table S1 Minimum inhibitory concentration (MIC) of AamAP1, AamAP1-Lys and GK-19 against five bacteria and three fungi.

Peptide / MIC (μM)	AamAP1	AamAP1-Lysine	GK-19
E.Coli	> 20	5	3
K. Pneumoniae	> 20	5	5
P. Aeruginosa	> 20	10	5
E. Faecalis	> 20	5	3
MRSA	> 20	5	5
C. Krusei	20	5	5
C. Albicans	> 20	10	10
C. Glabrata	> 20	10	10
